# Comparative Investigation of Combined Metabolomics-Flavoromics during the Ripening of Mango (*Mangifera indica* L.) cv. ‘Nam Dok Mai Si Thong’ and ‘Nam Dok Mai No. 4’

**DOI:** 10.3390/plants10102198

**Published:** 2021-10-16

**Authors:** Ye Lin Aung, Yaowapa Lorjaroenphon, Pinthip Rumpagaporn, Sudathip Sae-tan, Kriskamol Na Jom

**Affiliations:** Department of Food Science and Technology, Faculty of Agro-Industry, Kasetsart University, Bangkok 10900, Thailand; ye.a@ku.th (Y.L.A.); yaowapa.l@ku.th (Y.L.); pinthip.r@ku.th (P.R.); sudathip.sa@ku.th (S.S.-t.)

**Keywords:** metabolomics, flavoromics, mango, Nam Dok Mai, ripening, metabolites, flavors

## Abstract

A metabolomics-flavoromics approach was conducted to assess the micromolecules of ‘Nam Dok Mai Si Thong’ and ‘Nam Dok Mai No. 4’ mango cultivars from two seasons. During ripening, FAMEs, FFAs, fatty alcohols, sterols, and organic acids were dominant at 0–2 days, whereas amino acids, sugars, and volatile organic compounds, including esters, alcohols, ketones, aldehydes, and terpenes, were at higher levels at 4–8 days. Nine metabolites (palmitic/linoleic/linolenic/citric/malic acids, β-sitosterol, sucrose, glycine, and leucine) and two volatile organic compounds (ethyl octanoate/decanoate) were related to ripening-associated changes within eight days. During ripening, sucrose at 6–8 days, citric/malic acid at 0–2 days, glycine and leucine at 4 days, and ethyl octanoate and ethyl decanoate at 8 days could be used as quality biomarkers for Nam Dok Mai Si Thong; palmitic/linoleic/linolenic acids at 0 days and β-sitosterol at 0–4 days could be used as quality biomarkers for Nam Dok Mai No. 4.

## 1. Introduction

Mango (*Mangifera indica* L.) is one of the famous tropical fruits and is widely cultivated and consumed globally. Among the tropical climacteric fruits produced worldwide, mango production levels are second only to those of bananas [1]. Mango is held in high esteem, often being referred to as the ‘king of fruits’, a ‘heavenly fruit’ or a ‘superfruit’. The importance of the fruit arises from its nutritional quality, unique flavor, salubrious taste, and wholesomeness [2]. Mango is widely cultivated in Thailand; the Thai mango cultivar ‘Nam Dok Mai’ is well known and exported in large numbers to consumers worldwide who appreciate its nutritional value, specific texture, distinct flavor, and taste qualities [3]. Nam Dok Mai No. 4, Nam Dok Mai Si Thong, and ‘Mahachanok’ are popular cultivars among the exported mangoes from Thailand [4]. Nam Dok Mai Si Thong has an oval shape with a sharp, pointed tip and golden yellow color skin, and it is the major export mango variety in Thailand. It also has a sweet and scented taste with a very thin seed inside. Nam Dok Mai No. 4 has the same character as ‘Nam Dok Mai Si Thong’ but the influence of different genetic found by the skin color, which is still green when it is ripe. Of these, ‘Nam Dok Mai’ mango cultivars are one of the most popular export mangoes from Thailand, not only with consumers worldwide but also with the Thai people. Indeed, Nam Dok Mai cultivars are considered a favorite fruit in Thailand, particularly when presented with dessert rice.

Previous studies have indicated that morphological and molecular diversity analyses can facilitate mango cultivar identification [5,6]. However, it is also necessary to identify the biochemical features (i.e., the desired traits) of mangoes to supplement the data on the relationships among mango cultivars and to understand the functional aspects of the fruit. An appropriate experimental system should involve analysis of both the metabolomics and volatile organic compounds of the fruit, which would include analyses of an arrangement of chemicals from different classes, including lipids, acids, sugars, alcohols, aldehydes, esters, ketones, and terpenes [7].

To date, few omics-based studies have been published on the ripening process of mangoes. Moreover, combined metabolomics-flavoromics approaches have yet to be used for the analysis of ‘Nam Dok Mai’ mango cultivars. In the present study, we introduce a new application for rapid and reliable fingerprinting of volatile and non-volatile metabolites in mangoes; specifically, we combine metabolomics and flavoromics to track the ripening stage of ‘Nam Dok Mai Si Thong’ and ‘Nam Dok Mai No. 4’ in the dry and rainy seasons. Therefore, the aims of the study were to provide new insights into the ripening of ‘Nam Dok Mai’ mango cultivars by determining the relationships between ripening-related flavors and particular groups of metabolites as well as to identify potential biomarkers that indicate flavor quality according to mango ripening stage.

## 2. Results and Discussion

### 2.1. Ripening Appearance of ‘Nam Dok Mai’ Cultivars

**Figure 1:**
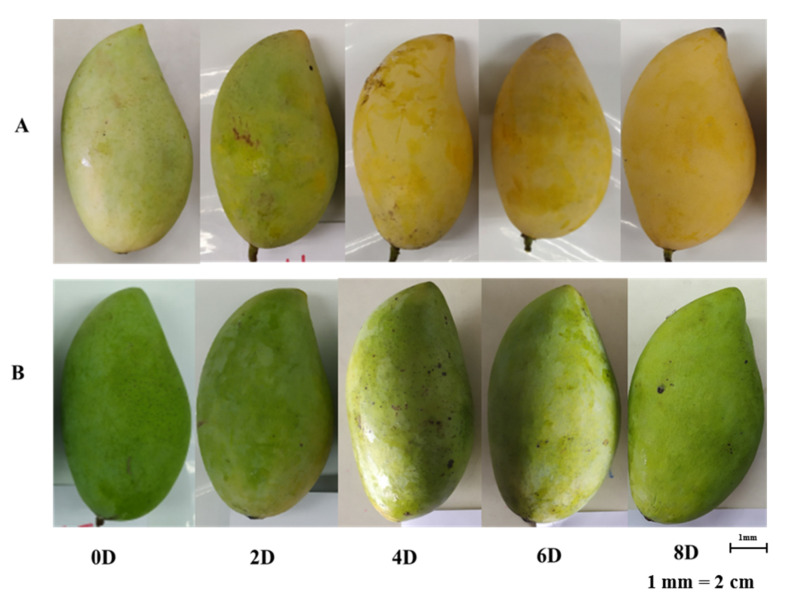
Appearance of Nam Dok Mai mango cultivars at different ripening stages. (**A**) ‘Nam Dok Mai Si Thon’; (**B**) ‘Nam Dok Mai No. 4 ‘(0 D to 8 D = 0 to 8 days of ripening).

Dry and rainy season mango samples were assessed at 0, 2, 4, 6, and 8 days of ripening. Color changes were observed from 0 to 8 days of ripening (Figure 1). The color of ‘Nam Dok Mai Si Thong’ mangoes was pale yellow to yellowish during the ripening process. However, ‘Nam Dok Mai No. 4’ mangoes had a green to pale green color over the same period, although the pulp color was altered. Chin et al. indicated that the color of ‘Chokanan’ mango cultivars changed from green to yellow within 0–8 days of ripening [8]. The degradation of pectin and the carbohydrate breakdown process occur during ripening. This is likely due to metabolic activities that can cause chemical changes, an increase in respiration, changes to structural polysaccharides causing softening, hydrolysis of starch into sugars, chlorophyll degradation, and carotenoid biosynthesis in mango fruit. Prasanna et al. indicated that a broad spectrum of biochemical changes occur during fruit ripening, such as biosynthesis of carotenoids, anthocyanins, essential oils, and flavor and aroma constituents, an increase in the activity of cell wall-degrading enzymes, and a transient increase in ethylene production [9].

### 2.2. Metabolite and Volatile Flavor Content of Ripening ‘Nam Dok Mai’ Mango Cultivars

In total, 98 metabolites were detected using GC-FID; 60% of 56 peaks with the highest and lowest quantitative affluence of metabolites were contributed by the ‘Nam Dok Mai Si Thong’ and ‘Nam Dok Mai No. 4’ cultivars. The metabolites of mango cultivars included 13 FAMEs, 20 polar lipids (free fatty acids, fatty alcohol, and phenols), 8 sugars (organic sugars and sugar alcohols), and 15 acids (organic and amino acids). Whereas volatile organic compounds with high vapor pressure were investigated using GC-ToF-MS with 48 total peaks including 35 identified compounds and 13 unidentified compounds comprising 8 esters, 13 alcohols, 5 ketones and aldehydes, 5 terpenes, 1 furan, 2 volatile acids, and 1 lactone. In the present study, micromolecules including lipophilics, hydrophilics, and high vapor pressure volatile organic compounds were analyzed by GC-FID and GC-Tof-MS. Previous studies evaluated in the way of cross-detector analysis by online coupling of gas chromatography (GC)-mass spectrometry (MS) and flame ionization detector (FID). GC-FID is useful for quantitative description and estimation of sample composition as well as molar ratios of different metabolites. On the other hand, GC-ToF-MS also provides reliable structural information with superior sensitivity. As a result, both detectors support a resilient tool for exploratory studies by supporting together with a powerful data-processing algorithm and could appear to be useful in metabolic profiling study [10]. However, the findings of Jumhawan et al. mentioned in coffee beans extracts by using GC-FID and GCMS application for creating metabolite identifying study. Gas chromatography/flame ionization detector (GC/FID) provided higher sensitivity over a homogenous scope of detected compounds than GC/MS [11]. Furthermore, Hübschmann also indicated that one of the common arguments against the routine use of the combination of FID and MS is the lack of complementarity. It has been assumed that MS and FID produced a resemble chromatogram, and response factors for the majority of organic compounds are comparable [12]. Previous studies reported that the conjugating of gas chromatography (GC) and liquid chromatography (LC) could be facilitated the selectivity of a wide range of compounds [13,14]. For the polar separation and analysis of metabolites, liquid chromatography gas chromatography (LCMS) has a more common separation for the polar analytes with larger molecules. LCMS can also be applied to separate any soluble compound, e.g., amino acids, proteins, drugs, nucleic acids, lipids, antioxidants, carbohydrates, and natural and artificial polymers. However, GC-FID could be easily detected for the separation and identification of small, volatile molecules, and it also has a higher sensitivity in the detection of polar compounds such as citric and malic acids. Similar findings were also reported in quality prediction of Asian palm civet coffee by using GC-FID technique [15]. Therefore, both techniques could provide the reliable detection and separation with high reproducibility for metabolite profiling. Moreover, coupling together with GC-FID and LCMS techniques for the separation and detection of metabolites should also be applied in the fields of food science among the distinct tropical fruit crops in the future.

**Figure 2:**
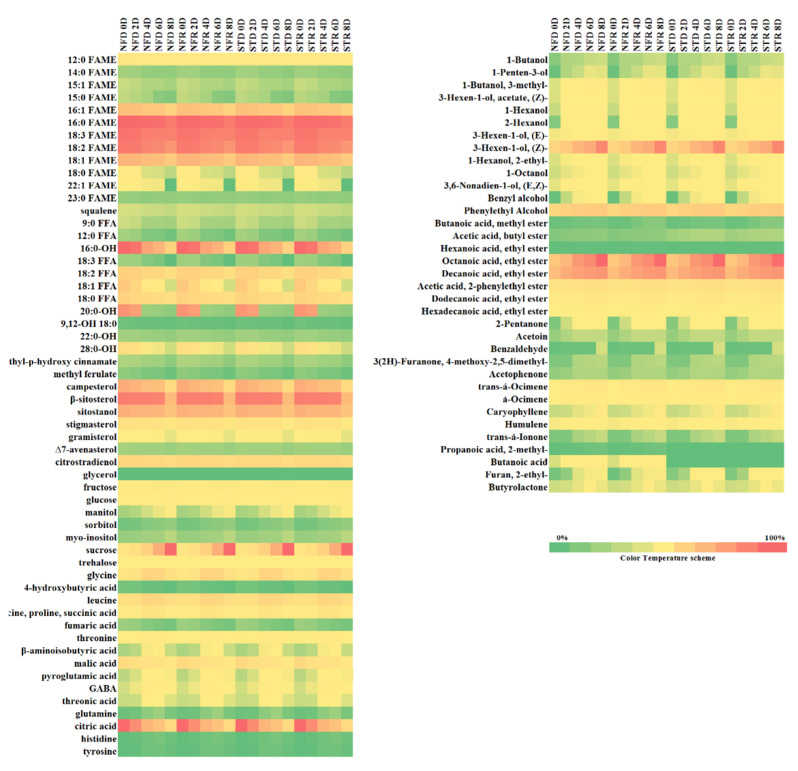
Heat plot of the metabolites and volatile organic compounds detected in dry and rainy season ‘Nam Dok Mai’ mangoes. FAME, fatty acid methyl ester; FFA, free fatty acid; GABA, gamma aminobutyric acid; STD, dry season ‘Nam Dok Mai Si Thong’; STR, rain season ‘Nam Dok Mai Si Thong’; NFD, dry season ‘Nam Dok Mai No. 4’; NFR, rainy season ‘Nam Dok Mai No. 4’. Red, yellow and green colors indicating higher, moderate and lower amounts, respectively.

A heat plot of two replicates normalized and standardized with an internal standard was also used to investigate more specific compounds during different ripening stages of the mango cultivars. Figure 2 presented the heat plot of metabolites and volatile organic compounds according to different ripening stages by cultivar and harvesting season.

#### 2.2.1. Lipid Fractions

Among the 13 FAMEs, 16:0 (palmitic acid), 16:1 (palmitoleic acid), 18:0 (stearic acid), 18:1 (oleic acid), 18:2 (linoleic acid), and 18:3 (linolenic acid) were selected because they showed dynamic fluctuations among the various lipid classes during days 0–8 of ripening. In addition, 16:0-OH, 22:0-OH, 18:2 free fatty acids (FFA), and 18:0 FFA were also found as primary fatty alcohols at 0 days of ripening. Their relative concentration gradually decreased as the fruit reached 8 days of ripening. Comparatively, the total lipid classes at 0 days were one-fold higher than those detected at 8 days of ripening in both cultivars and seasons. These fatty alcohols might be formed by reducing fatty acyl-CoAs or fatty acyl-ACPs catalyzed by a fatty acyl reductase [16]. Rowland et al. reported that NADPH-dependent fatty acyl reductases catalyze the development of fatty alcohols from fatty acyl-CoAs or fatty acyl-ACPs [17]. Additionally, Reineccius indicated that the largest variation of volatile organic compounds developed from lipids arises via lipoxygenase activity [18]. Fatty acids and lipids are relevant structural and metabolic components of plant and fruit cells; they often serve as precursors to important volatiles. For example, FFAs or those released by lipase activity and metabolized by β-oxidative enzymes and lipoxygenase are normally considered as the major forerunners of esters, alcohols, and aldehydes produced by fruits during development and maturation [19]. The major sterols detected in our analyses were β-sitosterol, campesterol, sitostanol, and citrostradienol; β-sitosterol and campesterol showed two-fold increases at the 0-day stage relative to at the 8-day stage, whereas sitostanol and citrostradienol levels were one-fold higher at 0 days than they were at 8 days. Concentrations were higher in the early and mid-ripe stages, especially in the dry season, for both ‘Nam Dok Mai’ mango cultivars, whereas total sterol content was more pronounced in ‘Nam Dok Mai No. 4’.

#### 2.2.2. Polar Fractions

To perform robustness and continuous injection, polar metabolites via derivatization reaction were also investigated using GC-FID. Changes in sugars and amino and acids were largely detected at the 4-, 6-, and 8-day ripening stages. Starch stored in the pulp during the early ripening stages of ‘Nam Dok Mai’ mango fruits might be due to the carbon source required for the soluble sugars synthesis. Consequently, the level of sugars substantially increased at 6 and 8 days of ripening. According to Sweetman et al., the metabolic pathway of gluconeogenesis, which results in the generation of glucose from phosphoenolpyruvate, appears mainly during fruit ripening when sugars compile promptly [20]. Additionally, Bernardes-Silva et al. found that the starch content of all cultivars of examined unripe mangoes had already decreased to about 50% and that the ripe fruit soluble sugar content reached 12% for ‘Haden’ and ‘Tommy’, 9.8% for ‘Palmer’, and 7.3% for ‘Van Dyke’ [21]. In the present investigation, the dominant sugar was sucrose, followed by fructose, glucose, and trehalose. The total sugar content was approximately 98% compared with other metabolites such as FAMEs, FFAs, fatty alcohols, and acids. These findings suggest that ‘Nam Dok Mai’ cultivars are rich sources of various sugars, including sucrose (23.6%–49.8%), fructose (1.3%–1.4%), glucose (0.7%–0.8%) and trehalose (~0.1%), at 6 and 8 days of ripening. Comparing the cultivars, ‘Nam Dok Mai Si Thong’ had ~1% more total sugar content than did ‘Nam Dok Mai No. 4’. Seasonal differences were apparent: dry season mangoes had slightly more sugars and sugar alcohols than did rainy season mangoes.

Amino acid content was significantly higher at day 4 of ripening in both cultivars. However, the amino acid content from 4 to 6 days of ripening varied only slightly. Both the ‘Nam Dok Mai’ cultivars had uniform acid content, although they were harvested in different seasons. The most prominent acids were glycine, leucine, and succinic acid, followed by threonine, β-aminoisobutyric acid, pyroglutamic acid, GABA, threonic acid, and fumaric acid. Glycine and leucine were significantly higher (one-fold increase) at the 4- and 6-day stages relative to their levels at the 0-, 2- and 8-day stages in both dry and rainy season mango cultivars. Consistently, succinic, fumaric, and α-ketoglutaric acid, which are the intermediates of the TCA cycle, also decreased as the ripening day increased. In contrast, as previously mentioned by Batista-Silva et al., the interrelation between organic acid and sugar metabolism during ripening seems to be a commonly associated aspect that contributes to the improvement of quality and flavor during fruit ripening [22]. Conversely, ‘Nam Dok Mai No. 4’ showed a slightly lower content of glycine, leucine, citric, and malic acid than did ‘Nam Dok Mai Si Thong’.

Citric and malic acids increased at 0 and 2 days of ripening by nearly six-fold (17.8% vs. 2.8%) and three-fold (2.8% vs. 1.06%), respectively, compared with their levels at 8 days of ripening. Comparing cultivars, the citric and malic acid levels were slightly higher in ‘Nam Dok Mai Si Thong’ than they were in ‘Nam Dok Mai No. 4’. Additionally, the dry season ‘Nam Dok Mai’ cultivars had slightly higher levels of citric and malic acids than were detected in the rainy season cultivars. Understanding the accumulation of these acids in fruit is of predominant to the priority of fruit quality. Citric acid and malic acid are generally recognized as safe-listed compounds that are commonly used in industry as preservatives, acidulants, or flavoring agents [23]; they are known to inhibit food spoilage and pathogenic microorganisms [24]. Furthermore, the concentration of citric and malic acids affects the sensorial and chemical characteristics of mango juice, e.g., pH, total acidity, microbial load, sweetness, and global acceptability. Previous studies have shown the influence of cultivation practices, along with irrigation [25,26], mineral fertilization [27,28] and thinning [25,29], and of environmental factors, such as temperature [30,31,32], on fruit acidity; however, it is unclear how these factors affect malic and citric acid accumulation.

#### 2.2.3. Volatile Organic Compounds (VOCs)

Even distribution of volatile organic compounds contents was observed in both dry and rainy season ‘Nam Dok Mai’ mango cultivars. Esters play an essential fraction of volatile organic compounds in many fruits [33]. At various ripening stages of both dry and rainy season ‘Nam Dok Mai’ cultivars, the major esters were octanoic acid, ethyl ester (4%–15%), decanoic acid, ethyl ester (5%–10%), acetic acid, 2-phenylethyl ester (1.1%–1.5%), dodecanoic acid and ethyl ester (0.4%–0.7%). From 0 to 8 days of ripening, the major alcohol volatiles in both dry and rainy season ‘Nam Dok Mai’ cultivars were 3-hexen-1-ol, (Z)- (4.9%–21%) followed by phenylethyl alcohol (4%–7%), 1-butanol, 3-methyl- (0.03%–0.08%), 3-hexen-1-ol, acetate, (Z)- (0.09%–0.27%) and 1-hexanol (0.07%–0.37%). Terpenes and aldehydes were slightly more prominent in ‘Nam Dok Mai Si Thong’. We found that the level of monoterpenes was higher than the level of sesquiterpenes. The major terpenes observed at different ripening stages were α-Ocimene (2%–4%), trans-α-Ocimene (2.8%–15.6%), caryophyllene (0.79%–10.4%), and humulene (1.1%–2.4%). Maximum content was detected at 6 and 8 days of ripening in both the dry and rainy season ‘Nam Dok Mai’ cultivars. Li et al. also demonstrated that monoterpenes were the most ample volatile constituents in mango germplasms from China, the Americas, Thailand, India, Cuba, Indonesia, and the Philippines [34]. Jiang and Song reported that amino acids are precursors for some branched aliphatic compounds [35]. These compounds can be further synthesized to configure esters, i.e., volatile compounds that provide many fruits their distinct ‘fruity’ odors. Brückner and Wyllie reported that the fruit volatile profiles were complicated and varied depending on the cultivar, ripeness, pre- and post-harvest environmental conditions, fruit sample (either intact fruit, slices, or homogenized samples), and analytical methods used [36].

In Thailand, the mango season occurs around mid-March to mid-June, which is usually regarded as the dry season. The rainy season is assumed to be from mid-July to mid-November. Dry season mangoes are usually harvested around March, whereas rainy season mangoes are usually picked around the end of June. Another critical factor to consider is that mangoes are produced all year round in Thailand. In the present study, mangoes harvested in the dry season showed slightly higher volatiles content. Comparing total volatile organic compounds between cultivars, dry season ‘Nam Dok Mai Si Thong’ was a rich source of esters, terpenes, aldehydes, and ketones, whereas dry season ‘Nam Dok Mai No. 4’ had slightly more total volatile alcohols than did ‘Nam Dok Mai Si Thong’. Considering the vital role played by these metabolites and aroma volatiles in imparting the characteristics of mango cultivars, ripe fruits of ‘Nam Dok Mai Si Thong’ and ‘Nam Dok Mai No. 4’ cultivars seem to possess differentiation and unique qualities with an abundance of metabolites and volatile organic compounds.

### 2.3. PCA

**Figure 3:**
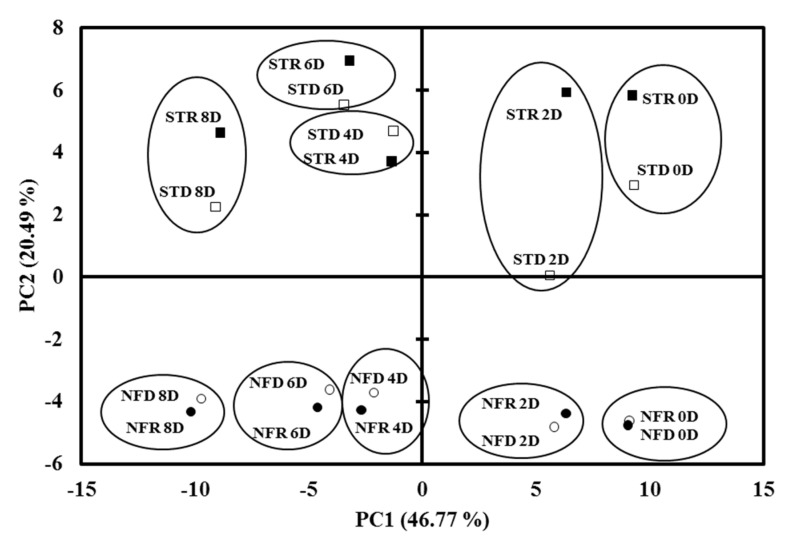
Principal component analysis of the combined metabolites at different ripening stages (0, 2, 4, 6, and 8 days of ripening) in mangoes. STD, dry season ‘Nam Dok Mai Si Thong’ (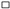); STR, rainy season ‘Nam Dok Mai Si Thong’ (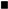); NFD, dry season ‘Nam Dok Mai No. 4’ (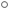); NFR, rainy season ‘Nam Dok Mai No. 4’ (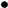).

The identified and obtained metabolites and volatiles data in dry and rainy season ‘Nam Dok Mai’ mango cultivars were clearly explored, and find the relationships between the samples by using PCA. Additionally, PCA is a multivariate data analysis method that can show the variance of samples using metabolites as descriptive data [37]. All metabolites were represented as 67% of total variables by PC1 and PC2. Figure 3 shows that genetic variation existed between the Nam Dok Mai mango cultivars. Indeed, the two ‘Nam Dok Mai’ cultivars were clearly differentiated by PC1 (20.49%). This indicates that there was an effect of the genetic variation of the two ‘Nam Dok Mai’ genotypes according to their metabolite content.

**Figure 4:**
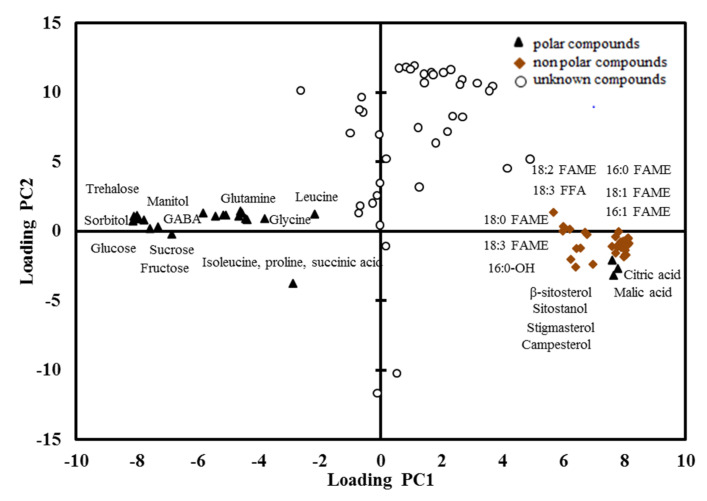
Loading plots of standardized GC/FID metabolite compounds (16:0 (palmitic acid), 16:1 (palmitoleic acid), 18:0 (stearic acid), 18:1 (oleic acid), 18:2 (linoleic acid), 18:3 (linolenic acid) and GABA (gamma aminobutyric acid) from the combined non-polar and polar fractions. Polar compounds (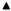); non-polar compounds (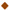); unknown compounds (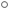). FAME, fatty acid methyl ester; FFA, free fatty acid; GABA, gamma aminobutyric acid.

Both ‘Nam Dok Mai’ cultivars were shifted along PC1 according to their ripening stage. Both dry and rainy season mango genotypes were typically distributed along PC2, depending on their quantity of metabolites. Their metabolite content also showed minor variation between seasons. Although there was a clear differentiation between the two genotypes along PC1, the metabolite compounds had similar concentrations in both ‘Nam Dok Mai’ cultivars. Therefore, the two ‘Nam Dok Mai’ mango cultivars had strong genetic and cultivar effects according to the quantitative abundance of their metabolites. This suggests that the major variability in metabolite content is given by the ripening status of the fruit, then by the genotype. The PCA loading plot was supported by observing the variance of metabolites, including the polar, non-polar, and unknown compounds analyzed in this study (Figure 4).

**Figure 5:**
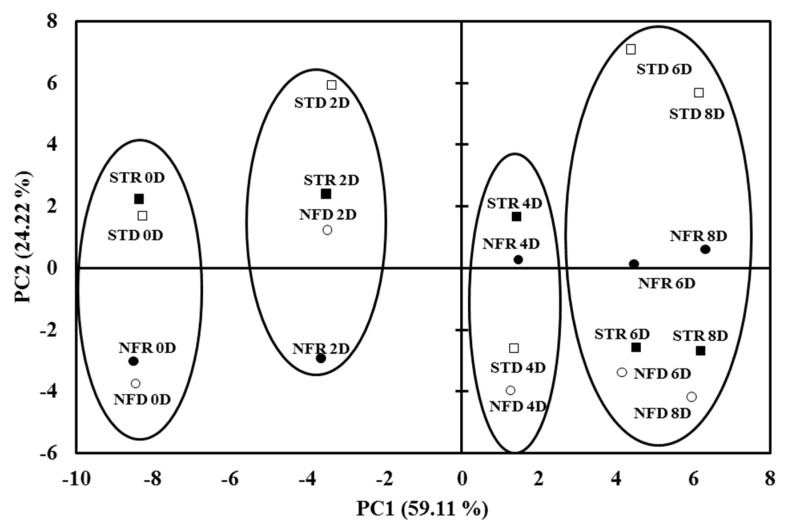
Principal component analysis of volatile organic compounds in the different ripening stages of mango fruit (0, 2, 4, 6, and 8 days (D)). STD, dry season ‘Nam Dok Mai Si Thong’ (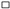); STR, rainy season ‘Nam Dok Mai Si Thong’ (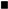); NFD, dry season ‘Nam Dok Mai No. 4’ (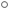); NFR, rainy season ‘Nam Dok Mai No. 4’ (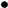).

**Figure 6:**
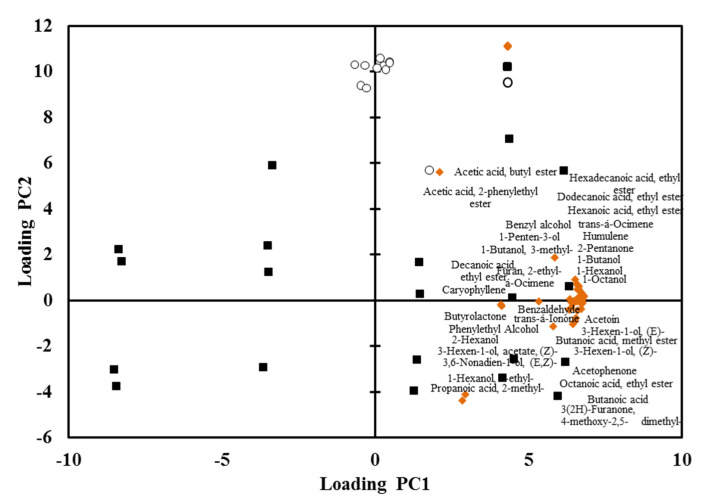
Loading plots of volatile organic compounds from the different ripening stages of mango fruits (0, 2, 4, 6, and 8 days of ripening). Volatile compounds (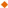); unknown compounds (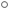); ripening stages (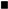).

Interestingly, most volatile organic compounds were equally variated in both PC1 and PC2, which represented 83% of the total variability (Figure 5). In PC2, both dry and rainy season ‘Nam Dok Mai Si Thong’ and ‘Nam Dok Mai No. 4’ were clearly distributed according to their volatile contents. For the ripening stage, the volatile content was also shifted along PC1. PCA loading plots also showed that a higher content of volatile organic compounds was apparent at the 6- and 8-day ripening stages in both ‘Nam Dok Mai’ cultivars (Figure 6). Low dependency was observed between cultivars and seasons in ‘Nam Dok Mai’ mango cultivars.

**Figure 7:**
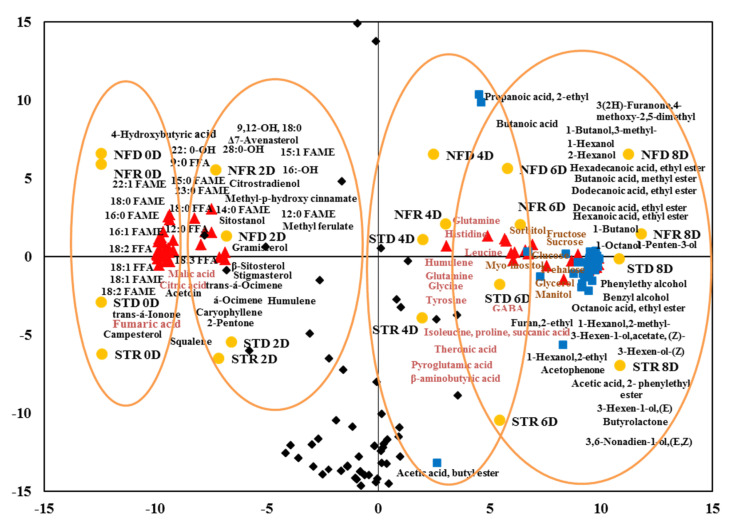
Principal component analysis (Biplot) from all metabolites (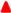) and volatile organic compounds (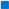), unknown metabolites and volatiles (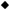) in ‘Nam Dok Mai’ mango cultivars (STD, dry season ‘Nam Dok Mai Si Thong’; STR, dry season ‘Nam Dok Mai Si Thong’; NFD, dry season ‘Nam Dok Mai No. 4’; NFR, rainy season ‘Nam Dok Mai No. 4’) (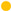). FAME, fatty acid methyl ester; FFA, free fatty acid; 0–8 D, 0–8 days of ripening.

The metabolites and organic volatile compounds showed four distinct clusters in a PCA biplot (Figure 7). The 0-day ripening stage of the dry and rainy season ‘Nam Dok Mai’ mangoes was situated in the first cluster group, whereas the second cluster group contained the 2-day ripened mangoes. FAMEs, FFA, fatty alcohols, sterols, and organic acids, especially citric acids, were abundant in the first and second clusters. The 4-day ripening stage comprised the third cluster group; most amino acids were found in this group. Sugars and volatile compounds were predominant in the fourth cluster group, which contained 6- and 8-day ripened dry and rainy season ‘Nam Dok Mai’ cultivars. Metabolites and volatile organic compounds were shifted along PC2, whereas different days of ripening (0–8 days) were represented by PC1. However, volatiles were also clearly separated according to different ripening stages in both dry and rainy season ‘Nam Dok Mai’ mango genotypes.

**Figure 8:**
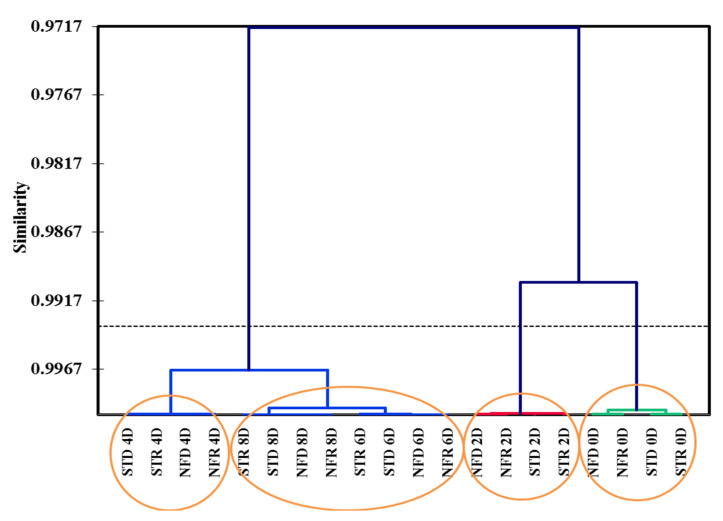
Agglomerative hierarchical clustering analysis of ‘Nam Dok Mai Si Thong’ mangoes from the dry and rainy season at different ripening stages (*p* ≤ 0.05). STD, dry season ‘Nam Dok Mai Si Thong’; STR, dry season ‘Nam Dok Mai Si Thong’; NFD, dry season ‘Nam Dok Mai No. 4’; NFR, rainy season ‘Nam Dok Mai No. 4’; 0–8 D, 0–8 days of ripening. Blue branch: fruits at 4 D, 6 D and 8 D at ripening stages, red branch: fruits at 2 D at ripening stages and green branch: fruits at 0 D ripening stage.

According to AHC analysis, a dendrogram was produced that contained four groups (Figure 8). The first group represented fruits at 0 days of ripening; the second group contained dry and rainy season ‘Nam Dok Mai’ cultivars at 2 days of ripening. The third group in the dendrogram contained 4-day ripened mangoes. Finally, the fourth group was subdivided into two subgroups containing the two mango cultivars within the dry and rainy season: (i) fruits at the 6-day ripening stage and (ii) those at the 8-day ripening stage. AHC analysis showed that the 4-day ripening stage was similar to the 6- and 8-day stages in both dry and rainy season ‘Nam Dok Mai Si Thong’ cultivars, whereas the 2-day ripening stage was closer to 0 days of ripening. This relationship likely reflects the metabolomic–flavoromic constituents that shifted among the various ripening stages of ‘Nam Dok Mai’ mango cultivars during ripening. In summary, changes to metabolites and volatile organic compounds were observed along the ripening stages, from 0 to 8 days, in ‘Nam Dok Mai’ mango cultivars. Although it was differentiated in the PCA biplot, AHC did not show differentiation of season to be genetics-based but rather due to ripening.

### 2.4. Correlation Analysis

**Figure 9:**
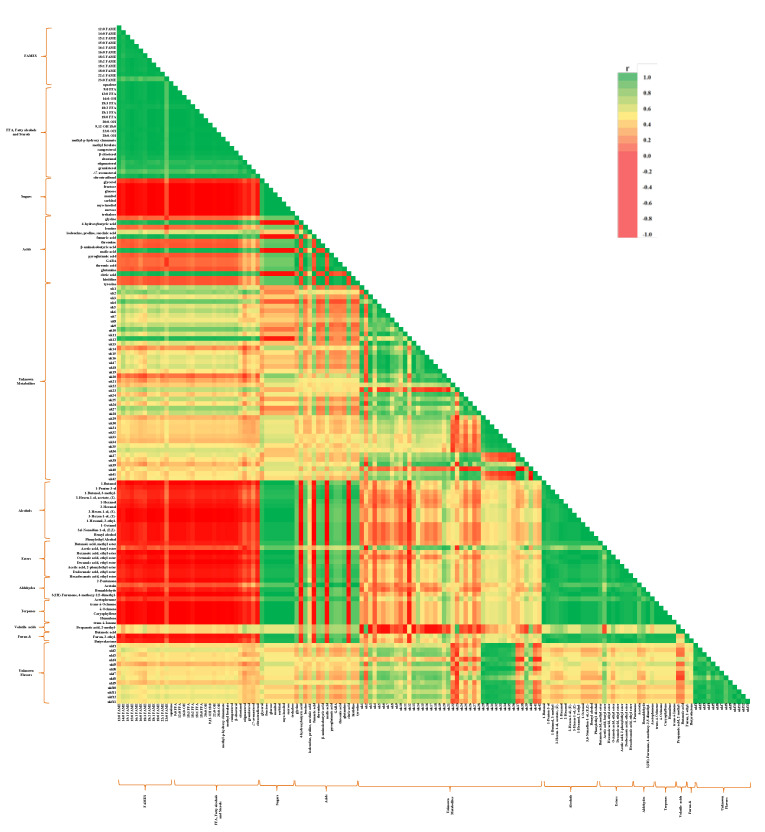
Pairwise triangular heat map correlation analysis among the metabolites and volatile organic compounds of mangoes at different ripening stages (*p* ≤ 0.05). Positive correlations (r > 0.3) with green color scale (r > 0.7 indicates a strong positive correlation). Negative correlations (r < −0.3) with red color scale (r < −0.7 indicates a strong negative correlation).

Pairwise correlation between metabolites was used to assess metabolic relationships among metabolites and the coordination of metabolic changes during the ripening of mangoes (Figure 9). According to the genetic similarity and minor seasonal variation between the two ‘Nam Dok Mai’ genotypes, metabolites and volatiles content showed equivalent distribution in the correlation analysis. However, significant correlations were observed based on the formation of compounds according to the different ripening stages. Lipophilic extracts, such as FAMEs, FFAs, fatty alcohols, free fatty acids, and sterols were slightly higher in ‘Nam Dok Mai No. 4’ and strongly correlated with the polar extracts citric and malic acid, fumaric acid, and 4-hydroxybutyric acid. Additionally, sugars, amino acids, and organic acids were relatively abundant in ‘Nam Dok Mai Si Thong’ and positively correlated with volatile organic compounds. As organic acids and sugars are critical components in the perception of mango fruit, ‘Nam Dok Mai Si Thong’ could be considered to possess a more attractive flavor than that of ‘Nam Dok Mai No. 4’.

As previously described by Wattanakul et al., amino acids play as the substrates for the formation of higher levels of alcohols and volatile acids as the result of the Ehrlich pathway [38]. Most FAMEs, FFAs, fatty alcohols, and sterols were strongly and negatively correlated with volatiles. In contrast, FAME and some amino acids such as glycine, leucine, succinic acid, threonine, histidine, and tyrosine resulted in no significant correlation between them. However, some of the unknown metabolite compounds were negatively correlated with volatile organic compounds. Therefore, the unknown metabolites from this study, as well as the volatile organic compounds, should be investigated further in subsequent research. The metabolite compounds detected in the present study are responsible for forming fragrant volatile organic compounds in the two mango cultivars during various ripening stages across two seasons. Therefore, the different ripening stages of ‘Nam Dok Mai’ mango cultivars would likely acquire high levels of bioactive compounds and desirable aroma compounds that would be favorable to further processing of mango products.

## 3. Materials and Methods

### 3.1. Chemicals and Reagents

Solvents and reagents, which were all of HPLC and analytical grade, were purchased from Thermo Fisher Scientific (Waltham, MA, USA). All standard chemicals, which were reagent grade, were purchased from Sigma-Aldrich (St. Louis, MO, USA). For the identification of metabolites, lauric acid methyl ester (12:0 FAME), myristic acid methyl ester (14:0 FAME), pentadecanoic acid methyl ester (15:0 FAME), pentadecenoic acid methyl ester 15:1 FAME, palmitic acid methyl ester (16:0 FAME), palmitoleic acid methyl ester (16:1 FAME), steric acid methyl ester (18:0 FAME), oleic acid methyl ester (18:1 FAME), linoleic acid methyl ester (18:2 FAME), linolenic acid methyl ester (18:3 FAME), erucic acid methyl ester (22:1 FAME), tricosanoic acid methyl ester (23:0 FAME), nonanoic acid (9:0 FFA), lauric acid (12:0 FFA), methyl-p-hydroxy cinnamate, methyl ferulate, hexadecanol (16:0-OH), steric acid (18:0 FFA), oleic acid (18:1 FFA), linoleic acid (18:2 FFA), linolenic acid (18:3 FFA), arachidyl alcohol (20:0-OH), linoleyl alcohol (9,12-OH 18:0), behenyl alcohol (22:0-OH), 1-octacosanol (28:0-OH), campesterol, β-sitosterol, sitostanol, stigmasterol, gramisterol, Δ7-avenasterol, citrostradienol, glycerol, fructose, glucose, mannitol, sorbitol, myo-inositol, sucrose, trehalose, glycine, 4-hydroxybutyric acid, leucine, isoleucine, proline, succinic acid, fumaric acid, threonine, β-aminoisobutyric acid, malic acid, pyroglutamic acid, γ- aminobutyric acid, threonic acid, glutamine, citric acid, histidine, and tyrosine were used as the authentic metabolite standards.

### 3.2. Fruit Selection and Ripening Condition

The mango samples were acquired from a Saraburi commercial orchard. Dry and rainy season mango samples were collected at 90–100 days after the fruit set and immediately transported to the laboratory at Kasetsart University, Bangkok, Thailand. Dry season samples were collected in mid-March, and rainy season samples were collected at the end of June. The fruits were selected according to their homogeneity in weight (400–420 g), length (14–16 cm), and width (7–9 cm). To control the ripening index, the uniform densities of the fruits were applied using 3% NaCl solution and sinkage in a 1% NaCl solution [39]. The selected samples were allocated in a controlled temperature storage chamber at 30 ± 2 °C and 75% ± 2% relative humidity to admit ripening. The samples were then taken after 0, 2, 4, 6, and 8 days of ripening.

### 3.3. Sample Preparation

After collecting the mango samples according to their days of ripening, the samples were washed, peeled, cut into 1 cm cubes, and freeze-dried in a Gamma 2-16 LSC freeze-drying machine (Martin Christ, Osterode am Harz, Germany). Freeze-dried samples were mill into a powder with an RS 300 rotor mill (Retsch, Haan, Germany). This powder was stored at −20 °C till it was analyzed.

### 3.4. Metabolomics

#### 3.4.1. Sample Extraction and Phase Separation

Extraction and fractionation of the samples were performed as previously described by Na Jom et al. [40] with a slight modification. The method of extraction covered a full range of lipophilic and hydrophilic low-molecular-weight compounds with two replications. The dried lipid fraction was re-dissolved in a combination of 500 µL methyl tert-butyl ether, 300 µL methanol, and 50 µL sodium-methylate for transesterification. Then, 1 mL dichloromethane and 2 mL 0.35 M hydrochloric acid solution were used to achieve selective hydrolysis. The 2 mL of 0.35 M hydrochloric acid solution was used to separate the upper phase. The lower phase, which included transmethylated lipids, was collected and dried by evaporation. With 250 µL of dichloromethane, the lipid residue was re-dissolved. Elution with varied ratios of hexane:methyl tert-butyl ether solution was used to fractionate transmethylated lipids, FAME, and polar lipids. Using a solid-phase microextraction C18-LP cartridge (VertiPak^TM^, C18-LP (Large Pore) Solid-phase extraction, Vertical Chromatography Co. Ltd.; Nonthaburi, Thailand), the ratio for fatty acid methyl esters was 100:2 *v*/*v* and 70:20 *v*/*v* for polar lipids. A parallel evaporator was used to evaporate all of the eluents at 50 °C. The fatty acid methyl ester (FAME) residue from the dried fraction was re-dissolved in 300 µL of hexane and kept in a glass amber vial. At 70 °C for 15 min, the dried fraction polar lipids were silylated with 250 µL pyridine and 50 µL N-trimethylsilyl-N-methyl trifluoroacetamide. Lipid fractions were stored at −20 °C until using gas chromatography-flame ionized detection (GC-FID) (Hewlett Packard, Palo Alto, CA, USA) analysis.

The 500 µL of polar extracts were evaporated till dry for the sugar fractionation. With 300 µL of pyridine and 100 µL of trimethylsilylimidazole, the polar residue was silylated. Silylation was carried out for 15 min in a water bath at 70 °C. Then, for the layer separation process, 300 µL of hexane and 300 µL of deionized water were added to separate the silylated sugars and sugar alcohol from the sample. The solution was then diluted with 300 µL of hexane, and the acid compounds were selectively hydrolyzed with 300 µL of deionized water. The GC-FID analysis was performed on the top layer of hexane, which contains silylated sugar compounds. The 1 mL of acids fractions was evaporated until dry with a parallel evaporator, then oximated with 300 µL of hydroxyl ammonium chloride in pyridine (2 mg/mL). After 30 min of oximation at 70 °C, silylation was performed by using 100 µL of N-methyl-N-(trimethylsilyl) fluoroacetamide to the reaction at 70 °C for 15 min. The 500 µL of hexane and 300 µL of deionized water were used for selective hydrolysis. The silylated sugars in the upper phase were eliminated. By adding 150 µL of acetonitrile and 100 µL of MSTFA at 70 °C for 60 min, the bottom phase, which contained acid compounds was re-dissolved and silylated. For GC-FID analysis, the sample was immediately stored at −20 °C. All polar and lipid extracts obtained were analyzed by gas chromatography with flame ionization detection (GC-FID).

#### 3.4.2. GC-FID Analysis

GC-FID analysis was performed by using gas chromatography (GC) conjoined with a flame ionization detector (Hewlett Packard, Palo Alto, CA, USA). The DB-1 capillary column (60 m × 0.32 mm × 0.25 μm film thicknesses) with a 100% dimethylpolysiloxane stationary phase (Agilent Technologies, Santa Clara, CA, USA) was used. Subsequently, 1 μL of each sample was injected into the GC flame ionization detector in splitless mode. As the carrier gas, helium was used at 1.8 mL per min with a constant flow rate. The inlet temperature was 280 °C; the oven temperature program started at 100 °C and then ramped up to 320 °C at 4 °C per min before being held at 320 °C for 25 min; the detector temperature was 320 °C.

### 3.5. Flavoromics

#### 3.5.1. Sample Extraction

Volatile organic compounds analysis followed the method described by Charve et al. [41]. A solid-phase microextraction (SPME) fiber (75-μm carboxen/polydimethylsiloxane) was used to extract volatiles from the headspace of mango puree solution. The fiber was exposed to the headspace of samples for 10 min at room temperature. SPME extraction was conducted under an incubation temperature of 50 °C with an extraction time of 20 min and with 0.3 g mL^−1^ of NaCl added to the samples. The samples were incubated at 50 °C for 5 min with constant magnetic stirring (60× *g*) before the SPME extraction process. The fiber was desorbed into the injection port of a gas chromatograph (260 °C) for 10 min. An inlet liner (78.5 × OD 6.5 × ID 0.75 mm) was used.

#### 3.5.2. GC-ToF-MS Analysis

Analyses were conducted using a 6890N gas chromatograph arrayed with an HP-5MS capillary column (30 m length, 0.25 mm id and 0.25 µm film thickness; J&W Scientific, Folsom, CA, USA) and paired with a time-of-flight mass spectrometer (Leco Corp., St. Joseph, MI, USA). The operating conditions were as follows: GC: split 5:1; oven temperature: initially 40 °C increasing by 5 °C per min to 150 °C and then 40 °C per min to 250 °C; TOF-MS: 40–280 u, centroid mode, 0.1 s scan time, 0.02 s inter-scan time, 2750 V and −70 eV. The GC chromatograms were compared using retention times, mass spectra of reference compounds, and mass spectra of library entries.

### 3.6. Statistical Analysis

Chromatographic areas of each metabolite were obtained and assimilated using the HP-ChemStation A.06.03 program (Hewlett Packard, Palo Alto, CA, USA). Flavor compounds were handled using ChromaTOF-GC Software v4.50.8.0 (Leco, St. Joseph, MI, USA). Identification involved using an analytical authentic standard comparison technique, and the process was semi-quantified using a comparative investigation with an internal standard area for metabolites and volatile organic compounds. Principal component analysis (PCA), agglomerative hierarchical clustering analysis (AHC), and Spearman’s rank correlations with a significance level of *p* ≤ 0.05 using the correlation/association test mode were performed using XLSTAT-base version 2020.5 (Addinsoft, NY, USA).

## 4. Conclusions

Here, a comparative investigation of combined metabolomics-flavoromics was conducted that provided new insights into the different ripening stages of two ‘Nam Dok Mai’ cultivars across two seasons. Mangoes from the dry and rainy seasons were highly similar with approximately equal loads of various micromolecules. Sucrose at 6–8 days, citric and malic acid at 0–2 days, glycine and leucine at 4 days, and ethyl octanoate and ethyl decanoate at 8 days of ripening could be used as quality biomarkers for ‘Nam Dok Mai Si Thong’. In contrast, palmitic, linoleic, and linolenic acid at 0 days and β-sitosterol at 0–4 days of ripening could be used as quality biomarkers for ‘Nam Dok Mai No. 4’. In conclusion, ‘Nam Dok Mai Si Thong’ and ‘Nam Dok Mai No. 4’ cultivars could show substantial genetic viability across cultivars and seasons by demonstrating an equivalent distribution of metabolomics-flavoromics constituents at various ripening stages. The integration of combined metabolomics-flavoromics approach provided a useful tool applied for characterizing the remarkable alterations pointing out biomarker compounds that occur in ‘Nam Dok Mai’ mango cultivars during different ripening stages within the dry and rainy seasons.

## Data Availability

The data that support the findings of this study are accessible on request from the corresponding author. The data are not publicly available due to privacy or ethical restrictions.
